# Caspase-independent cell death does not elicit a proliferative response in melanoma cancer cells

**DOI:** 10.1186/s12860-018-0164-1

**Published:** 2018-07-04

**Authors:** Ahlima Roumane, Kevin Berthenet, Chaïmaa El Fassi, Gabriel Ichim

**Affiliations:** 1 INSERM 1052, CNRS 5286, Cancer Research Center of Lyon (CRCL), Lyon, France; 2 LabEx DEVweCAN, Université de Lyon, Lyon, France

**Keywords:** Apoptosis, Caspase-independent cell death, Compensatory proliferation

## Abstract

**Background:**

Apoptosis, the most well-known type of programmed cell death, can induce in a paracrine manner a proliferative response in neighboring surviving cells called apoptosis-induced proliferation (AiP). While having obvious benefits when triggered in developmental processes, AiP is a serious obstacle in cancer therapy, where apoptosis is frequently induced by chemotherapy. Therefore, in this study, we evaluated the capacity of an alternative type of cell death, called caspase-independent cell death, to promote proliferation.

**Results:**

Using a novel in vitro isogenic cellular model to trigger either apoptosis or caspase-independent cell death, we found that the later has no obvious compensatory proliferation effects on neighboring cells.

**Conclusions:**

This study enforces the idea that alternative types of cell death such as caspase-independent cell death could be considered to replace apoptosis in the context of cancer treatment.

**Electronic supplementary material:**

The online version of this article (10.1186/s12860-018-0164-1) contains supplementary material, which is available to authorized users.

## Background

Cancer aggressiveness is the sum of resistance to therapy, enhanced metastatic potential and intense proliferation of cancer cells. Seminal studies performed in the 1950s revealed an odd paradigm: lethally irradiated cancer cells boost the proliferation of neighboring, healthy cells [1]. This paradoxical effect was tackled few years ago when dying cells were shown to elicit a wound healing response in neighboring cells intended to rapidly cope with tissue destruction. This so-called apoptosis-induced proliferation or AiP was described in several organisms such as flies, Hydra or Xenopus [2, 3]. Briefly, in *Drosophila melanogaster*, apoptotic cells secrete several mitogens such as Wingless (Wnt analogue) or Decapentaplegic (Dpp, similar to TGF-β) that stimulate AiP. Even more striking, Hydra can regenerate its entire head and this is completely prevented by caspase inhibition. Similarly, the Xenopus tadpole can re-grow its tail following amputation. The molecular mechanisms are broadly discussed in recent reviews [2–4].

In mouse, dying cells can equally affect their neighbouring cells. In an elegant study, Fang Li and colleagues found that dying fibroblasts could potentiate the proliferation of different stem cell lines [5]. In a more physiological setting, this had a positive effect on wound healing and even liver regeneration [5]. Mechanistically, the authors provide strong evidence for the effector caspases 3 and 7-driven production of prostaglandin E_2_ (PGE_2_), a powerful proliferative signal molecule [6, 7]. Collectively, these studies highlight the PGE_2_ pathway as a rigorously conserved mechanism that triggers immediate cell proliferation to cope with dangerous tissue destruction.

This so-called “Phoenix rising” effect is now established in different types of cancers such as melanoma, glioma and pancreatic ductal adenocarcinoma [8–10]. How can PGE_2_ have such a dramatic effect on tumour repopulation following an apparently successful cancer treatment? Developmental studies in Zebra fish and mice bring in vivo evidence for the cooperation between PGE_2_ and Wnt3 in hematopoietic stem cells (HSC) expansion and organ regeneration where PGE_2_ is required for phosphorylation-mediated β-catenin protein stability [11]. The role of PGE_2_ in the “Phoenix rising” effect certainly relies on additional functions. Along this line, PGE_2_ orchestrates various processes such as HSC mobilization, angiogenesis by promoting endothelial cell migration via ERK or by sustaining and facilitating dendritic cells migration through MMP9 induction [12–14].

Cancer is known to hijack for its own proliferative advantage several survival pathways and PGE_2_ seems to be one extra addition to its toolbox.

Since apoptosis can trigger the AiP of adjacent cancer cells, it is therefore imperative to find alternative types of cell death that could be induced by cancer therapy while lacking the proliferation-inducing effect. Apoptosis might not be the best therapeutic choice for several other reasons. First, the non-lethal caspase activation resulting from incomplete mitochondrial outer-membrane permeabilisation (MOMP) could induce DNA damage, genomic instability and ultimately favors tumourigenesis [4, 15–17]. Moreover, classical death receptors such as CD95 and TRAIL receptors could also paradoxically promote oncogenesis. Sub-lethal doses of TRAIL or CD95 ligand were shown to induce DNA damage and genetic mutations in a caspase-8 and CAD dependent manner [18].

Surprisingly, recent findings demonstrate that CD95/CD95L support growth of tumour cells via basal activation of JNK while tissue specific deletion of CD95 reduces the incidence of liver and endometrioid ovarian cancer [19]. CD95 is also responsible for the metastatic spread in glioblastoma, gastrointestinal cancer and pancreatic adenocarcinoma. This is achieved by increased MMP secretion and EMT induction [20–22]. The death ligand TRAIL also plays a double role: it can specifically kill cancer cells, however it was also proposed to have an oncogenic role. Accordingly, TRAIL treatment enhances the inflammatory phenotype and metastasis of pancreatic and breast cancer cells [23, 24]. Moreover, mutant K-RAS switches both TRAIL and CD95L from potent death ligands into pro-metastatic migration cues [25]. A more recent study reveals that TRAIL-R drives proliferation and migration through Rac1/PI3K axis. Furthermore, high human TRAIL-R2 expression is a factor of bad prognosis in patients with mutant K-RAS cancers [26]. A broader perspective was adopted by Tubio and Estivill who argues that recovery from apoptosis can be one of the mechanisms responsible for chromothripsis by which a single genetic event leads to hundreds of genomic rearrangements [27–29].

Caspase-independent cell death or CICD might be a potential candidate for such an anti-cancer alternative therapeutic strategy. CICD is basically apoptosis lacking caspase activation and therefore its kinetics are much slower compared to apoptosis. Caspase inhibition can be ensured by treatment with chemical caspase inhibitors such as Q-VD-OPh, Z-VAD-fmk or by ectopically expressing caspase inhibitor proteins such as XIAP or p35 [30]. Recently, CICD gained attention as a promising replacement for apoptosis in cancer treatment when Giampazolias et al. demonstrated that CICD triggers via NF-kB a sustained pro-inflammatory cytokine storm leading to enhanced anti-tumoural activity compared to apoptosis [31, 32]. In this research article we set up a novel in vitro system in melanoma cancer cells to trigger either apoptosis or CICD and compare the paracrine proliferation-inducing effect that might be triggered by either type of cell death and found that CICD lacks the capacity to increase the proliferation of surviving cancer cells.

## Results

### Setting up a cellular model to specifically trigger apoptosis or CICD

**Fig. 1:**
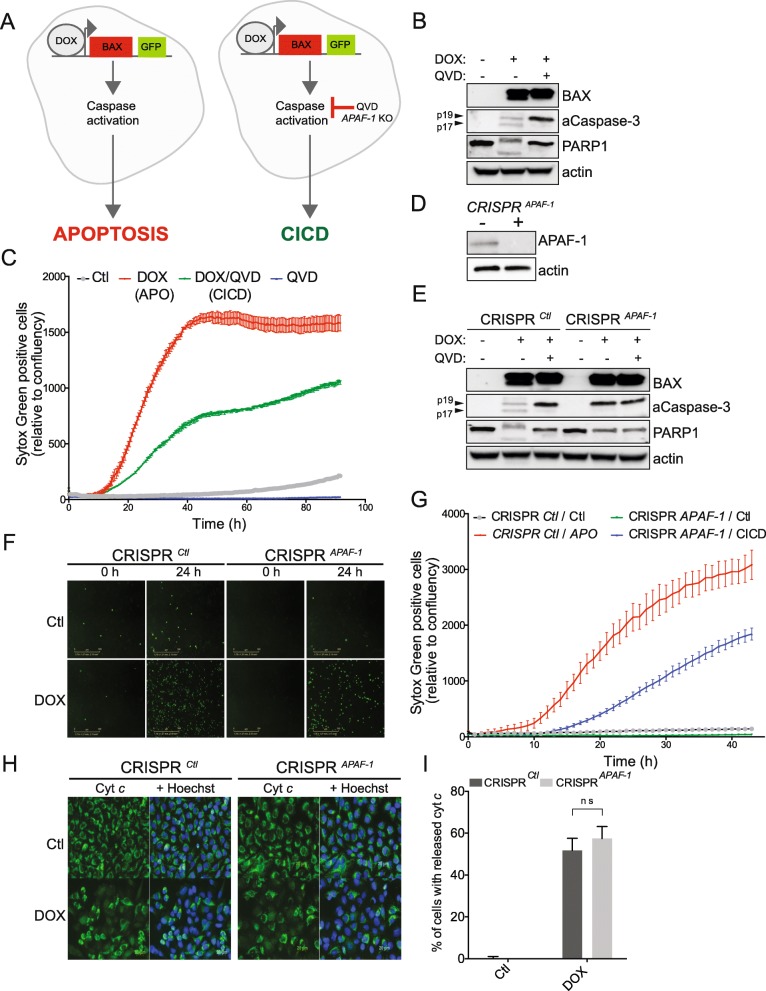
Efficient engagement of either apoptosis or CICD in melanoma cells. **a**. In vitro system of triggering either apoptosis or CICD in melanoma cells based on doxycycline-mediated rapid expression of the pro-apoptotic BAX protein. **b** Apoptosis (1 μg/ml DOX-treated cells) or CICD (DOX and Q-VD-OPh treated cells) was engaged in WM115 for 24 h followed by immunoblotting for BAX, caspase-3, PARP1 and actin as loading control. **c** WM115 cells were treated as in (**b**) and cell viability was measured by SYTOX Green exclusion in an Incucyte Imager. A representative experiment is shown. **d** Efficacy of CRISPR/Cas9-mediated *APAF-1* KO in WM115 cells. **e** Validation of apoptosis and CICD induction in *APAF-1* KO WM115 cells. DOX treatment and immunoblotting was done as described in (**b**). **f** Representative SYTOX Green positive staining for either apoptotic or cells undergoing CICD at 24 h. **g** Cell death kinetics for apoptosis and CICD triggered in the context of *APAF-1* KO. A representative experiment is shown. **h-i**. Immunofluorescence representative images (**h**) for the release of cytochrome *c* and quantification (**i**)

Following mitochondrial permeabilisation, cells are sentenced to certain death: when caspases are activated, this occurs within minutes through classical apoptosis and if caspase function is blocked, cells will equally die through CICD although with a considerately slower kinetic. In order to compare the proliferation triggered by both apoptosis and CICD and evaluate their therapeutic benefits, we first set up an in vitro model to specifically trigger either apoptosis or CICD. For this, we used melanoma cancer cells (WM115 and 501Mel) in which we stably expressed a doxycycline-inducible *BAX* transgene. Of note, BAX is a potent pro-apoptotic protein that efficiently triggers MOMP. In order to engage CICD, caspase activation was blocked either by chemical inhibition using the pan-caspase inhibitor Q-VD-OPh or by CRISPR/Cas9-mediated knockout of *APAF-1*, which is critical for caspase activation (Fig. 1a). First, we assessed by western blotting the expression of BAX following doxycycline (DOX) treatment. As shown in Fig. 1b, doxycycline (DOX) treatment leads to increased BAX expression, with processing of caspase-3 while we also observed cleavage of PARP1, indicative of apoptosis initiation. Importantly, the co-treatment with Q-VD-OPh prevents caspase activation (Fig. 1b). Of note, the addition of Q-VD-OPh prevents the maturation of caspase-3 subunit p19 into the fully active p17, which explains the lack of PARP1 cleavage and inhibition of apoptosis under these conditions (Fig. 1b). Next, we took advantage of the live-cell Incucyte Imager system to evaluate the induction of either apoptosis or CICD in BAX-expressing melanoma cells. Figure 1c shows that BAX expression rapidly triggers apoptosis as evidenced by SYTOX Green exclusion. In contrast, CICD (Q-VD-OPh-treated cells) has a slower kinetic (Fig. 1c). To circumvent any potential side effect of using Q-VD-OPh, we took advantage of CRISPR/Cas9-mediated gene editing to delete *APAF-1* and therefore abrogate caspase activation (Fig. 1d for KO efficacy). This was tested in more details in Fig. 1e which shows that *APAF-1* deletion efficiently blocks caspase-3 activation and PARP1 cleavage. Regarding the effect on cell death, triggering cell death in the context of *APAF-1* deletion equally induces CICD (Fig. 1f and g). This was further validated in 501Mel melanoma cell line (Additional file 1: Figure S1A-C). The release of cytochrome *c* from mitochondria is a well-established hallmark of MOMP. In our system, both apoptosis and CICD are characterized by the same percentage of cytochrome *c* release 24 h after DOX treatment (Fig. 1h and i). Moreover, the CICD triggered in our in vitro system is characterized by secretion of several cytokines as shown in Additional file 1: Figure S1 D and E, as described recently [31, 32]. Overall, these results validate our in vitro model for triggering either apoptosis or CICD.

### Apoptosis triggers proliferation in neighboring cells while CICD does not

**Fig. 2:**
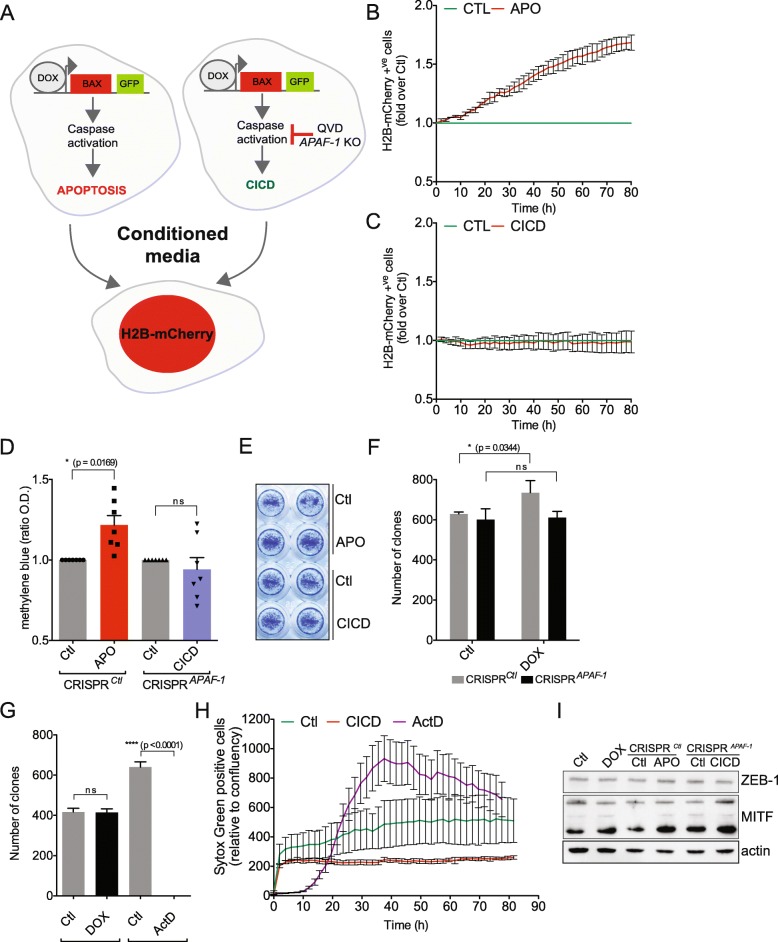
CICD does not trigger proliferation of melanoma cancer cells. **a** Working model for testing the effect of apoptotic and CICD conditioned media on the proliferation of neighboring cells. **b-c** Incucyte analysis for the proliferation of WM115 H2B-mCherry cells grown in the presence of APO (**b**) or CICD (**c**) conditioned media, obtained 24 h after triggering either apoptosis or CICD. *n* = 4–5 independent experiments; mean values +/− s.e.m. **d** Same as in (**b-c**), while this time cell proliferation was assessed by quantifying the optical density (O.D.) of methylene blue staining of cells grown in either apoptotic or CICD media. **e** Representative images of methylene blue staining. **f** Compensatory proliferation tested and quantified by clonogenic survival assay performed using the same conditions described in (**b**). **g** Parental WM115 cells were incubated with doxycycline (1 μg/ml) and the conditioned media was then added on H2B-mCherry expressing WM115 cells while clonogenic survival was assessed. Actinomycin D (ActD) treatment was used as negative control. **h** Incucyte analysis for the cell death induction (SYTOX Green exclusion) triggered by CICD conditioned media in WM115 H2B-mCherry cells. Actinomycin D treatment (1 μM) is used as positive control for cell death induction. A representative experiment is shown. **i** Immunoblotting for ZEB-1-MITF transcription factors axis of WM115 cells grown in APO and CICD conditioned media for 48 h. Actin was used as loading control

Since engaging CICD might replace apoptosis in anti-cancer therapy, we next addressed the issue of proliferation-inducing effects that might be generated by either type of cell death. For this, we triggered apoptosis or CICD in WM115 cells by DOX-mediated BAX expression and 24 h later the conditioned media was added on WM115 cells stably expressing mCherry-tagged H2B histone to better quantify cell proliferation using the Incucyte Imager (Fig. 2a). We chose the 24 h time point since the levels of complete mitochondrial permeabilisation, a point of no return for cell survival, were comparable between apoptosis and CICD (Fig. 1i). Importantly, while apoptotic conditioned media promotes the proliferation of H2B-mCherry WM115 cells, the CICD does not, with even a slight inhibition of cell proliferation observed in some experiments (Fig. 2b and c). Moreover, in agreement with previously published research, we found that under apoptotic conditions, the WM115 cells secrete PGE_2_ while as expected this is not the case for the melanoma cells undergoing CICD (Additional file 2: Figure S2A, B). The production of PGE_2_ is COX-2-dependent since the use of celecoxib, an inhibitor of COX-2, abrogates PGE_2_ secretion. In a functional assay, we also confirmed that apoptotic conditioned media obtained in the presence of celecoxib lacks the AiP effect (Additional file 2: Figure S2 C).

We also confirmed the lack of proliferation triggered by CICD by either methylene blue staining of surviving cells (Fig. 2d, e), clonogenic survival (Fig. 2f, g) or simply by counting the H2B-mCherry WM115 cells (Additional file 3: Figure S3A). The same controls were done for doxycycline alone to excluded any interference of the drug with cell proliferation (Fig. 2g, Additional file 3: Figure S3B-D). We equally confirmed that CICD lacks AiP in a second melanoma cell line, the WM239A (Additional file 3: Figure S3E-H). This effect is not hindered by excessive apoptosis since CICD conditioned media did not trigger apoptosis in WM115 or WM239A H2B-mCherry cells as shown by the SYTOX Green exclusion kinetic in Fig. 2h and Additional file 3: Figure S3I. The master regulator of melanoma cell proliferation is the MITF/ZEB-1 couple of transcription factors. To test whether they are involved in the effect that both apoptosis and CICD have on proliferation, we assessed the expression of both MITF and ZEB-1 by western blotting following the incubation with conditioned media, however we did not find any major changes in their expression (Fig. 2i). The same was found for the NF-kB pathway, another signaling hub that could affect proliferation. More specifically, NF-kB activation was measured by assessing NF-kB p65 nuclear translocation in WM115 cells incubated with either apoptotic of CICD conditioned media, while treatment with TNFα was used as positive control (Additional file 3: Figure S3J).

We next addressed whether this contrasting effect on proliferation of neighboring cells is not influenced by the differences in actual cell death. For this we obtained conditioned media from apoptotic or CICD cells at either 24 or 40 h, respectively, when the extent of cell death was comparable between the two conditions (Additional file 4: Figure S4A). In accordance with our previous results, CICD still lacks the capacity to trigger the proliferation of either WM115 (Additional file 4: Figure S4 B-E) or WM239A cells (Additional file 4: Figure S4 F-I).

Together, these results provide an important insight into the capacities of both apoptosis and CICD to drive the potentially dangerous proliferation of neighboring cancer cells, which then might favor cancer relapse.

### CICD delays cancer cell migration

**Fig. 3:**
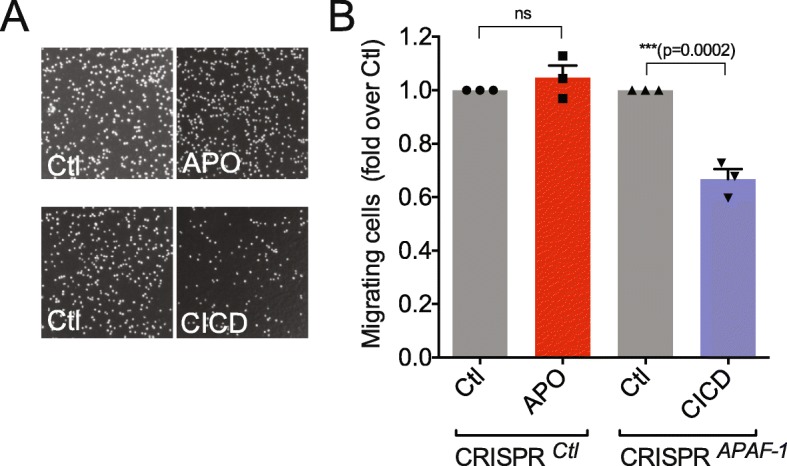
Impact of apoptosis and CICD on melanoma cell migration. **a** Hoechst 33342 nuclear staining for migrating WM115 cells through the 8-μm transwell membranes. Prior to the transwell assay, the WM115 cells were grown in APO or CICD conditioned media for 48 h. **b** Quantification of transwell assay performed on WM115 cells cultured either in APO or CICD conditioned media as in (**a**). *n* = 3 independent experiments; mean values +/− s.e.m.

Increased cancer cell migration is a major contribution to cancer aggressiveness. We therefore aimed to understand whether growing WM115 H2B-mCherry cells in either apoptotic or CICD media for 48 h could impact on their capacity to migrate. First, apoptotic conditioned media does not impart any migratory advantage to melanoma cells as assessed by transwell assay (Fig. 3a and b). Surprisingly, CICD significantly reduced transwell migration (Fig. 3a and b). While additional migration and invasion test are needed in support, these findings hint that CICD might also delay cancer cell migration.

## Discussion

We generated here an in vitro system in melanoma cells to evaluate the capacity of either apoptosis or CICD to trigger proliferation in neighboring cancer cells. This is based on the inducible expression of BAX in cells proficient or deficient for caspase activation, which allows efficient induction of either apoptosis or CICD. The use of the pan-caspase inhibitor Q-VD-OPh or CRISPR/Cas9-mediated *APAF-1* deletion ensures effective inhibition of caspase activation. It is worth paying attention however to the use of Q-VD-OPh. This inhibitor blocks with higher specificity the maturation of caspase-3, preventing the conversion of p19 into p17 subunit and therefore it prevents full activation of caspase-3 [33]. This is also the case when using CRISPR/Cas9—mediated gene editing to silence *APAF-1* (Fig. 1b, e). This explains the accumulation of the p19 subunit, which in our model can neither trigger apoptosis nor mediate the release of PGE_2_. We also cannot exclude that TNF-α produced under CICD conditions might trigger the activation of caspase-8 in neighboring cells, leading to partially activated caspase-3 [32]. Nevertheless, we admit that intermediate p19 subunit could still be active in certain physiological settings [34]. For instance, the p19/p12 complex regulates microglia activation via a PKCδ-dependent pathway [35].

Nonetheless, this system allowed us to reassert that indeed apoptosis promotes cell proliferation, while surprisingly CICD lacks this effect. This is of particular interest for cancer therapy since engaging apoptosis could in some settings favor the oncogenic process [4, 36]. AiP is most probably due to the release of PGE_2_, a derivate of the arachidonic acid, which was recently identified as a pro-tumorigenic factor in several cancers, mainly due to its pro-proliferative function [36]. Pharmacological inhibition of PGE_2_ pathway in cancer is still in pre-clinical stage, however the results are so far encouraging. A recent study showed that dying cells release PGE_2_ that elicit a “wound healing” gene response in cancer stem cells (CSC) driving their proliferation and eventually tumour repopulation. Importantly, co-treatment with celecoxib (a selective inhibitor of COX-2 that drives PGE_2_ synthesis) or a PGE_2_ neutralizing antibody abrogates CSCs proliferation [37]. COX2 inhibitors are definitely promising yet, on their own, they are far from being the silver bullet for cancer therapy. A 2014 meta-analysis of 11 clinical trials using celecoxib in combination with chemo-, radio- or hormone therapy revealed a significant increase in the overall response rate with no effect however on 1-year mortality. Moreover, celecoxib increases the risk of cardiovascular complications [38]. These studies further support the idea that in the context of cancer therapy, triggering apoptosis might not be the best choice, at least in certain circumstances.

The novelty of the present study comes from our evidence that CICD is in stark contrast with apoptosis regarding the proliferative response triggered in a paracrine manner in neighboring cells. Although we could not determine the underlying mechanisms, we could speculate on this. Unlike apoptosis, CICD is characterized by a NF-kB-dependent production of several cytokines, some of them having an anti-proliferative potential, at least in melanoma. The CICD-mediated cytokine storm is very similar to the senescence-associated secretory phenotype (SASP). Interestingly, chronic exposure to SASP can induce a bystander senescence effect in neighboring cancer cells [39]. Being limited to in vitro observations this study lacks insights into the in vivo complexity of tumors where the immune, stromal and vasculature cells might behave differently in the presence of CICD-triggered cytokine storm. Of interest, Giampazolias and colleagues recently argue that engaging CICD often leads to complete tumor regression by triggering a sustained anti-tumoral immune response [31, 32].

More broadly, research is now needed to determine the best approaches to trigger CICD in the context of cancer treatment. One possibility would be using pan-caspase inhibitors together with the classical chemotherapy. Orally available pan-caspase inhibitors were tested in mouse models of non-alcoholic fatty liver disease (NAFLD) with promising decrease in hepatocytes cell death, reduction of inflammation and lower oxidative stress [40]. In a phase-2 trial on patients with non-alcoholic steatohepatitis, inhibition of caspase-1, − 8 and − 9 using the GS-9450 compound significantly improved liver function [41]. A concern for using caspase inhibitors in vivo would be that blocking cell death might promote tumourigenesis, however a study evaluating the long-term exposure to emricasan, a pan-caspase inhibitor, revealed that its use was not carcinogenic at all [42].

One of the most significant findings to emerge from this study is that CICD completely lacks in vitro the capacity to induce the proliferation of surviving cancer cells, making it a promising alternative to apoptosis when envisaging novel therapeutic approaches.

## Methods

### Cells

All cell lines were maintained in DMEM high glucose medium supplemented with 10% FBS, 2 mM glutamine, 1 mM sodium pyruvate, non-essential aminoacids and penicillin/streptomycin.

### Plasmid constructs

The tetON piTR1 HA-BAX and pQXIN H2B-mCherry plasmids were a gift from David Goldschneider and Luca Fava, respectively. CRISPR/Cas9 *APAF-1* lentiviral plasmid was obtained by ligating the sgRNA ACAGCCTGCCATTCCATGTA into the lentiCRISPR V2 (Addgene 52961).

### Lentiviral transduction

293 T cells (2 × 10^6^ in a 10 cm dish) were transfected with lentiCRISPR V2 *APAF-1* together with the packaging plasmids using Lipofectamine 3000 (Life Technologies) according to the manufacturer’s instructions. Two days later virus-containing supernatant was harvested, filtered and used to infect WM115 and 501Mel cells in the presence of 1 μg/ml of polybrene. Two days post-infection, stably expressing cells were selected by growth in blasticidin.

### Immunofluorescence

WM115 cells were cultured on coverslips then fixed for 10 min in 4% PFA and permeabilised in PBS/0.2% Triton. Cells were then incubated for 1 h with blocking solution (2% BSA in PBS) and then with the appropriate primary antibody (1 h or over-night). The following antibodies were used: anti-cytochrome *c* (Cell Signaling, 12963) and p65 (Cell Signaling, 6956S). After 3 washes in PBS, cells were incubated for 1 h with the respective secondary antibody and Hoechst 33342. Images were obtained by fluorescence microscopy (Zeiss microscope) and analyzed using Adobe Photoshop and ImageJ.

### Cytokine antibody microarray

The CICD-conditioned media was analysed using the Human Cytokine Antibody Array (Abcam, ab133997) according to the manufacturer’s instructions.

### Transwell in vitro migration assays

6 × 10^4^ WM115 cells were placed in the upper chamber of 8 μm Transwells (Corning, CLS3422-48EA) in serum-depleted media, while the lower chamber was filled with FBS-supplemented culture media (20%). 24 h later the cells on top of the membrane were cleansed with a cotton swab while the cells that migrated on the other side were stained with Hoechst 33342 and counted.

### Western blotting

Cell lysates were prepared using NP-40 lysis buffer (1% NP-40, 1 mM EDTA, 150 mM NaCl, 50 mM Tris pH 7.4, 1 mM PMSF, Complete Protease Inhibitors [Roche]). Protein content was determined by Bio-Rad assay, proteins were separated by SDS-PAGE and blotted onto nitrocellulose. Membranes were probed with antibodies at 1/1000 dilution unless otherwise stated. BAX (Santa Cruz, sc-493), caspase-3 (Cell Signalling, 9662), PARP1 (eBioscience, 14–6666), actin-HRP (Sigma-Aldrich, A3854), APAF-1 (Merk, AB16941), ZEB-1 (Sigma-Aldrich, HPA027524), MITF (Abcam, ab20663) antibodies followed by incubation with the appropriate HRP conjugated secondary antibody and detection by ECL.

### Incucyte imager-based cell viability and proliferation assays

Cell viability was determined using an Incucyte Zoom imaging system (Essen Bioscience). Cells were plated in medium containing 30 nM SYTOX Green (Life Technologies, S7020). Cells were then treated with doxycycline (1 μg/ml), actinomycin D (1 μM) or conditioned media from either apoptotic or CICD cells and then imaged every 60 or 120 min. The analysis was done using Incucyte image analysis software (Essen Bioscience). For the proliferation assays, the H2B-mCherry expressing cells were incubated with either apoptotic or CICD media and the number of H2B-mCherry positive nuclei was automatically counted over time. For quantification, the number of SYTOX Green or H2B-mCherry positive cells was normalized to the initial confluency factor of the respective well.

### Clonogenic survival assay

For the clonogenic survival assay, 800 cells were plated into each well of a 6-well tissue culture plate. The following day, cells were treated with the indicated stimuli. Ten days later, the colonies were stained with 1% methylene blue in methanol/H_2_O (1:1 vol/vol) Colonies were counted using Fiji software (http://fiji.sc/Fiji) and compared with untreated cells.

### ELISA assay

Apoptotic or CICD conditioned media obtained in the presence or absence of celecoxib (5 μM) was harvested, centrifuged at 2500 g for 5 min and stored at − 80 °C until use. PGE_2_ production was determined using the PGE_2_ high sensitivity ELISA kit (Enzo Life Sciences, Cat. No. ADI-930-001) according to the manufacturer’s instructions.

### Statistical analysis

For comparison of multiple groups, two-way Analysis of Variance (ANOVA) was used while Student’s t test was applied when comparing two groups. Analyses were performed using Prism 5.0 software (GraphPad).

## Conclusions

To conclude, our results strongly indicate that an alternative type of cell death, called caspase-independent cell death (or CICD), is preferable to apoptosis regarding cancer treatment since it does not trigger proliferation of surrounding cancer cells. Moreover, it seems that CICD impairs in a paracrine manner melanoma cells migration. However, further investigations are needed to better characterise the underlying mechanisms and test whether our observations have any clinical significance for cancer therapy.

## Additional files

Additional file 1: **Figure S1.** (related to Fig. 1). A. Immunoblotting validation of CRISPR/Cas9-mediated *APAF-1* KO in 501Mel cells. Actin is used as loading control. B. Cell death kinetics for apoptosis and CICD triggered by doxycycline treatment in tetON BAX expressing 501Mel. A representative experiment is shown. C. Representative SYTOX Green positive staining for either apoptotic or cells undergoing CICD at 24 h in tetON BAX expressing 501Mel cells. D. Cytokine antibody array immunoblotting for CICD conditioned media. tetON BAX-expressing *APAF-1* KO WM115 cells were treated with DOX (1 μg/ml) for 24 h then the conditioned media was processed for the cytokine antibody microarray. E. Relative quantification of selected cytokines in CICD conditioned media. For D and E, data is presented from one representative experiment (out of two). (PDF 461 kb)

Additional file 2: **Figure S2.** (related to Fig. 2). A-B. Detection of PGE_2_ by ELISA in apoptotic (A) or CICD (B) conditioned media obtained in the presence or absence of celecoxib (5 μM). C. Incucyte analysis for the proliferation of WM115 H2B-mCherry cells grown in apoptotic conditioned media obtained in the presence or absence of celecoxib (5 μM). *n* = 4 independent experiments; mean values +/− s.e.m. (PDF 142 kb)

Additional file 3: **Figure S3.** (related to Fig. 2). A. WM115 H2B-mCherry cell counting following 48 h of incubation with either apoptotic or CICD conditioned media. B. Cell counting for assessing the potential effect of doxycycline treatment on WM115 H2B-mCherry cells. C. Cell proliferation of WM115 H2B-mCherry cells in response to doxycycline treatment (1 μg/ml) was assessed by quantifying the optical density of methylene blue staining. D. Same as in C, representative methylene blue-stained cells are shown. E-F. Incucyte analysis for the proliferation of WM239A H2B-mCherry cells grown in the presence of APO (E) or CICD (F) conditioned media. *n* = 3 independent experiments; mean values +/− s.e.m. G-H. Cell proliferation of WM239A H2B-mCherry cells grown for 48 h in APO or CICD conditioned media was measured by quantifying the optical density (O.D.) of methylene blue staining (G), while in H same approach was done to exclude a potential effect of DOX treatment on cell proliferation. I. Incucyte analysis for the cell death induction (SYTOX Green exclusion) triggered by CICD conditioned media in WM239A H2B-mCherry cells. Actinomycin D treatment (1 μM) is used as positive control for cell death induction. A representative experiment is shown. J Immunofluorescence representative images for the nuclear translocation of p65 in WM115 H2B-mCherry cells grown in presence of apoptotic or CICD conditioned media. Treatment for 3 h with TNFα (20 ng/ml) was used as positive control for p65 nuclear translocation. (PDF 706 kb)

Additional file 4: **Figure S4.** (related to Fig. 2). A. SYTOX Green analysis for the cell death kinetics allowing to identify the 24 and 40 h time frame where apoptosis and CICD, respectively, reach the same extent of execution. A representative experiment is shown. B-C. WM115 H2B-mCherry cells were grown in either apoptotic (obtained at 24 h following doxycycline treatment) (B) or CICD conditioned media (collected 40 h after triggering CICD) (C) and the number of H2B-mCherry positive cells was automatically counted using an Incucyte imager. n = 3 independent experiments; mean values +/− s.e.m. D-E. Representative images of methylene blue staining (D) and the optical density quantification (E) for assessing the proliferation of WM115 H2B-mCherry cells grown as described in B-C. F-I. Same as in B-E, the proliferation of WM239A H2B-mCherry cells is determined by either Incucyte imager or methylene blue staining. For the Incucyte analysis, *n* = 4–5 independent experiments; mean values +/− s.e.m. (PDF 329 kb)

## Abbreviations

APAF-1: Apoptotic protease activating factor 1
BAX: BCL2-associated X protein
CAD: Caspase-activated DNase
Cas9: CRISPR-associated protein 9
CD95: Cluster of differentiation 95
CICD: Caspase-independent cell death
CLX: Celecoxib
COX-2: Cyclooxigenase 2
CRISPR: Clustered regularly interspaced short palindromic repeats
CSC: Cancer stem cell
DOX: Doxycycline
Dpp: Decapentaplegic
EMT: Epithelial-mesenchymal transition
GFP: Green fluorescent protein
H2B: Histone 2B
HSC: Hematopoietic stem cells
JNK: Jun N-terminal kinase
MITF: Melanogenesis associated transcription factor
MMP: Matrix metalloproteinase
MOMP: Mitochondrial outer-membrane permeabilisation
NAFLD: Non-alcoholic fatty liver disease
NF-kB: Nuclear factor kappa-light-chain-enhancer of activated B cells
PARP: Poly(ADP-ribose) polymerase
PGE_2_: Prostaglandin E_2_
SASP: Senescence-associated secretory phenotype
TGF-β: Transforming Growth Factor Beta
TRAIL: TNF-related apoptosis-inducing ligand
Wnt: Wingless-related integration site
XIAP: X-linked inhibitor of apoptosis protein
ZEB1: Zinc finger E-box-binding homeobox 1

## References

[1] Revesz L (1956). Effect of tumour cells killed by x-rays upon the growth of admixed viable cells. Nature.

[2] Fuchs Y, Steller H (2015). Live to die another way: modes of programmed cell death and the signals emanating from dying cells. Nat Rev Mol Cell Biol.

[3] Ryoo HD, Bergmann A (2012). The role of apoptosis-induced proliferation for regeneration and cancer. Cold Spring Harb Perspect Biol.

[4] Ichim G, Tait SW (2016). A fate worse than death: apoptosis as an oncogenic process. Nat Rev Cancer.

[5] Li F, Huang Q, Chen J, Peng Y, Roop DR, Bedford JS, Li CY (2010). Apoptotic cells activate the "phoenix rising" pathway to promote wound healing and tissue regeneration. Sci Signal.

[6] North TE, Goessling W, Walkley CR, Lengerke C, Kopani KR, Lord AM, Weber GJ, Bowman TV, Jang IH, Grosser T (2007). Prostaglandin E2 regulates vertebrate haematopoietic stem cell homeostasis. Nature.

[7] Castellone MD, Teramoto H, Williams BO, Druey KM, Gutkind JS (2005). Prostaglandin E2 promotes colon cancer cell growth through a Gs-axin-beta-catenin signaling axis. Science.

[8] Donato AL, Huang Q, Liu X, Li F, Zimmerman MA, Li CY (2014). Caspase 3 promotes surviving melanoma tumor cell growth after cytotoxic therapy. J. Invest. Dermatol.

[9] Mao P, Smith L, Xie W, Wang M (2013). Dying endothelial cells stimulate proliferation of malignant glioma cells via a caspase 3-mediated pathway. Oncol Lett.

[10] Cheng J, Tian L, Ma J, Gong Y, Zhang Z, Chen Z, Xu B, Xiong H, Li C, Huang Q (2015). Dying tumor cells stimulate proliferation of living tumor cells via caspase-dependent protein kinase Cdelta activation in pancreatic ductal adenocarcinoma. Mol Oncol.

[11] Goessling W, North TE, Loewer S, Lord AM, Lee S, Stoick-Cooper CL, Weidinger G, Puder M, Daley GQ, Moon RT (2009). Genetic interaction of PGE2 and Wnt signaling regulates developmental specification of stem cells and regeneration. Cell.

[12] Hoggatt J, Mohammad KS, Singh P, Hoggatt AF, Chitteti BR, Speth JM, Hu P, Poteat BA, Stilger KN, Ferraro F (2013). Differential stem- and progenitor-cell trafficking by prostaglandin E2. Nature.

[13] Rao R, Redha R, Macias-Perez I, Su Y, Hao C, Zent R, Breyer MD, Pozzi A (2007). Prostaglandin E2-EP4 receptor promotes endothelial cell migration via ERK activation and angiogenesis in vivo. J Biol Chem.

[14] Yen JH, Khayrullina T, Ganea D (2008). PGE2-induced metalloproteinase-9 is essential for dendritic cell migration. Blood.

[15] Cartwright IM, Liu X, Zhou M, Li F, Li CY (2017). Essential roles of Caspase-3 in facilitating Myc-induced genetic instability and carcinogenesis. Elife.

[16] Ichim G, Lopez J, Ahmed SU, Muthalagu N, Giampazolias E, Delgado ME, Haller M, Riley JS, Mason SM, Athineos D (2015). Limited mitochondrial permeabilization causes DNA damage and genomic instability in the absence of cell death. Mol Cell.

[17] Liu X, He Y, Li F, Huang Q, Kato TA, Hall RP, Li CY (2015). Caspase-3 promotes genetic instability and carcinogenesis. Mol Cell.

[18] Lovric MM, Hawkins CJ (2010). TRAIL treatment provokes mutations in surviving cells. Oncogene.

[19] Chen L, Park SM, Tumanov AV, Hau A, Sawada K, Feig C, Turner JR, Fu YX, Romero IL, Lengyel E (2010). CD95 promotes tumour growth. Nature.

[20] Kleber S, Sancho-Martinez I, Wiestler B, Beisel A, Gieffers C, Hill O, Thiemann M, Mueller W, Sykora J, Kuhn A (2008). Yes and PI3K bind CD95 to signal invasion of glioblastoma. Cancer Cell.

[21] Zheng HX, Cai YD, Wang YD, Cui XB, Xie TT, Li WJ, Peng L, Zhang Y, Wang ZQ, Wang J (2013). Fas signaling promotes motility and metastasis through epithelial-mesenchymal transition in gastrointestinal cancer. Oncogene.

[22] Teodorczyk M, Kleber S, Wollny D, Sefrin JP, Aykut B, Mateos A, Herhaus P, Sancho-Martinez I, Hill O, Gieffers C (2015). CD95 promotes metastatic spread via Sck in pancreatic ductal adenocarcinoma. Cell Death Differ.

[23] Trauzold A, Siegmund D, Schniewind B, Sipos B, Egberts J, Zorenkov D, Emme D, Roder C, Kalthoff H, Wajant H (2006). TRAIL promotes metastasis of human pancreatic ductal adenocarcinoma. Oncogene.

[24] Fritsche H, Heilmann T, Tower RJ, Hauser C, Von Au A, El-Sheikh D, Campbell GM, Alp G, Schewe D, Hubner S (2015). TRAIL-R2 promotes skeletal metastasis in a breast cancer xenograft mouse model. Oncotarget.

[25] Hoogwater FJ, Nijkamp MW, Smakman N, Steller EJ, Emmink BL, Westendorp BF, Raats DA, Sprick MR, Schaefer U, Van Houdt WJ (2010). Oncogenic K-Ras turns death receptors into metastasis-promoting receptors in human and mouse colorectal cancer cells. Gastroenterology.

[26] von Karstedt S, Conti A, Nobis M, Montinaro A, Hartwig T, Lemke J, Legler K, Annewanter F, Campbell AD, Taraborrelli L (2015). Cancer cell-autonomous TRAIL-R signaling promotes KRAS-driven cancer progression, invasion, and metastasis. Cancer Cell.

[27] Tubio JM, Estivill X (2011). Cancer: when catastrophe strikes a cell. Nature.

[28] Stephens PJ, Greenman CD, Fu B, Yang F, Bignell GR, Mudie LJ, Pleasance ED, Lau KW, Beare D, Stebbings LA (2011). Massive genomic rearrangement acquired in a single catastrophic event during cancer development. Cell.

[29] Forment JV, Kaidi A, Jackson SP (2012). Chromothripsis and cancer: causes and consequences of chromosome shattering. Nat Rev Cancer.

[30] Tait SW, Green DR (2008). Caspase-independent cell death: leaving the set without the final cut. Oncogene.

[31] Giampazolias E, Tait SWG (2017). Caspase-independent cell death: an anti-cancer double-whammy. Cell Cycle.

[32] Giampazolias E, Zunino B, Dhayade S, Bock F, Cloix C, Cao K, Roca A, Lopez J, Ichim G, Proics E (2017). Mitochondrial permeabilization engages NF-kappaB-dependent anti-tumour activity under caspase deficiency. Nat Cell Biol.

[33] Han Z, Hendrickson EA, Bremner TA, Wyche JH (1997). A sequential two-step mechanism for the production of the mature p17:p12 form of caspase-3 in vitro. J Biol Chem.

[34] Pop C, Salvesen GS (2009). Human caspases: activation, specificity, and regulation. The Journal of Biological Chemistry.

[35] Kavanagh E, Rodhe J, Burguillos MA, Venero JL, Joseph B (2014). Regulation of caspase-3 processing by cIAP2 controls the switch between pro-inflammatory activation and cell death in microglia. Cell Death Dis.

[36] Fogarty CE, Bergmann A (2017). Killers creating new life: caspases drive apoptosis-induced proliferation in tissue repair and disease. Cell Death Differ.

[37] Kurtova AV, Xiao J, Mo Q, Pazhanisamy S, Krasnow R, Lerner SP, Chen F, Roh TT, Lay E, Ho PL (2015). Blocking PGE2-induced tumour repopulation abrogates bladder cancer chemoresistance. Nature.

[38] Chen J, Shen P, Zhang XC, Zhao MD, Zhang XG, Yang L (2014). Efficacy and safety profile of celecoxib for treating advanced cancers: a meta-analysis of 11 randomized clinical trials. Clin Ther.

[39] Schosserer M, Grillari J, Breitenbach M (2017). The dual role of cellular senescence in developing tumors and their response to cancer therapy. Front Oncol.

[40] Anstee QM, Concas D, Kudo H, Levene A, Pollard J, Charlton P, Thomas HC, Thursz MR, Goldin RD (2010). Impact of pan-caspase inhibition in animal models of established steatosis and non-alcoholic steatohepatitis. J Hepatol.

[41] Ratziu V, Sheikh MY, Sanyal AJ, Lim JK, Conjeevaram H, Chalasani N, Abdelmalek M, Bakken A, Renou C, Palmer M (2012). A phase 2, randomized, double-blind, placebo-controlled study of GS-9450 in subjects with nonalcoholic steatohepatitis. Hepatology.

[42] Elbekai RH, Paranjpe MG, Contreras PC, Spada A (2015). Carcinogenicity assessment of the pan-caspase inhibitor, emricasan, in Tg.rasH2 mice. Regul Toxicol Pharmacol.

