# Supplementary figures and images for: Caspase-independent cell death does not elicit a proliferative response in melanoma cancer cells
Source: BMC Cell Biol. 2018 Jul 4;19:11. doi: 10.1186/s12860-018-0164-1 (PMC6030751; doi:10.1186/s12860-018-0164-1)

Figure S1

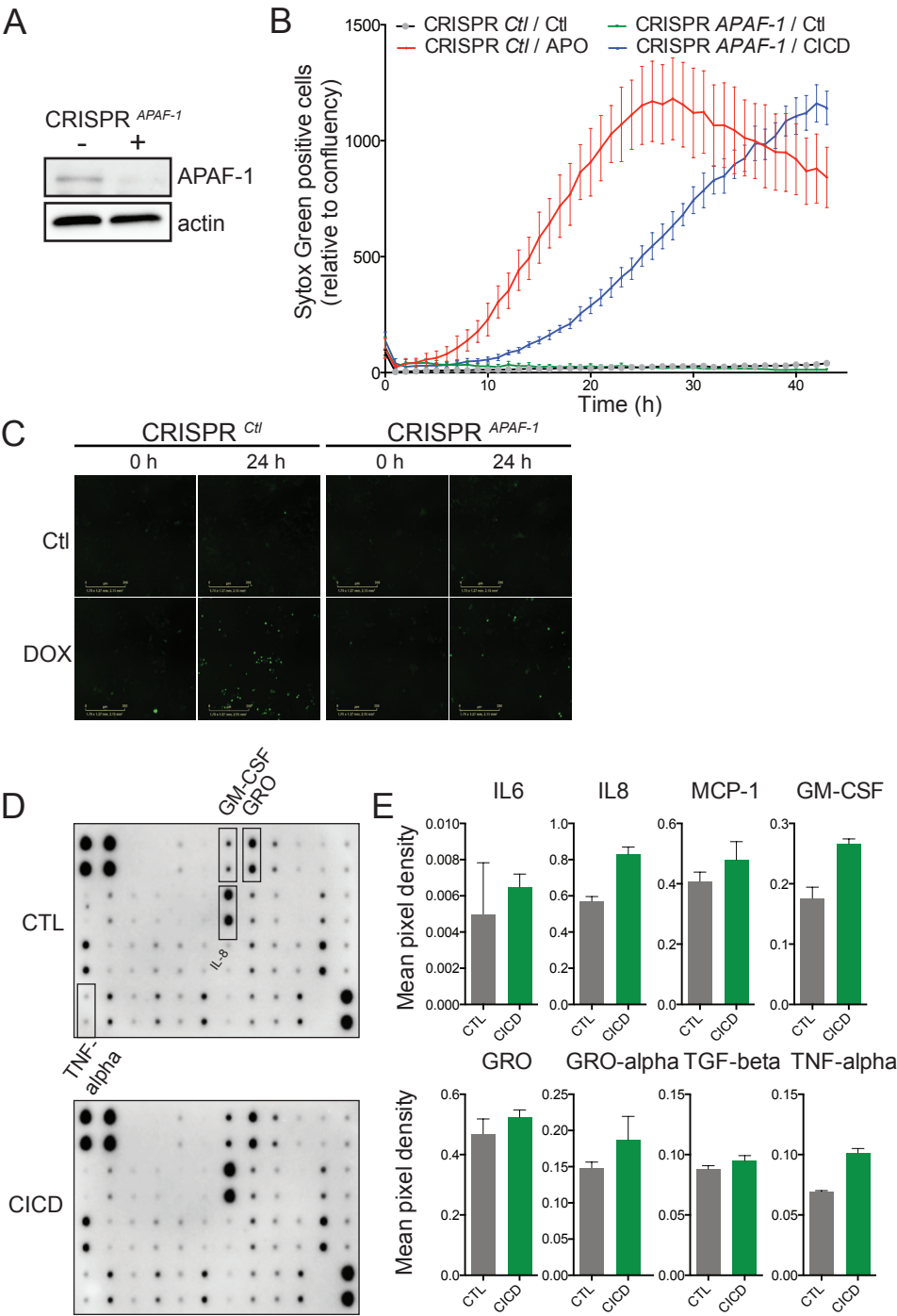

Supplement: Supplementary file 1 — Figure S1. (related to Fig. 1). A. Immunoblotting validation of CRISPR/Cas9-mediated APAF-1 KO in 501Mel cells. Actin is used as loading control. B. Cell death kinetics for apoptosis and CICD triggered by doxycycline treatment in tetON BAX expressing 501Mel. A representative experiment is shown. C. Representative SYTOX Green positive staining for either apoptotic or cells undergoing CICD at 24 h in tetON BAX expressing 501Mel cells. D. Cytokine antibody array immunoblotting for CICD conditioned media. tetON BAX-expressing APAF-1 KO WM115 cells were treated with DOX (1 μg/ml) for 24 h then the conditioned media was processed for the cytokine antibody microarray. E. Relative quantification of selected cytokines in CICD conditioned media. For D and E, data is presented from one representative experiment (out of two). (PDF 461 kb) [file 12860_2018_164_MOESM1_ESM.pdf]

Figure S2

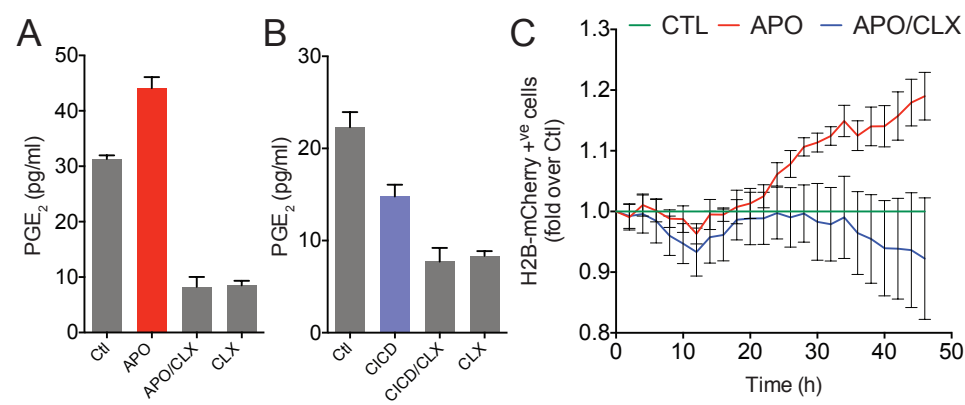

Supplement: Supplementary file 2 — Figure S2. (related to Fig. 2). A-B. Detection of PGE2 by ELISA in apoptotic (A) or CICD (B) conditioned media obtained in the presence or absence of celecoxib (5 μM). C. Incucyte analysis for the proliferation of WM115 H2B-mCherry cells grown in apoptotic conditioned media obtained in the presence or absence of celecoxib (5 μM). n = 4 independent experiments; mean values +/− s.e.m. (PDF 142 kb) [file 12860_2018_164_MOESM2_ESM.pdf]

Figure S3

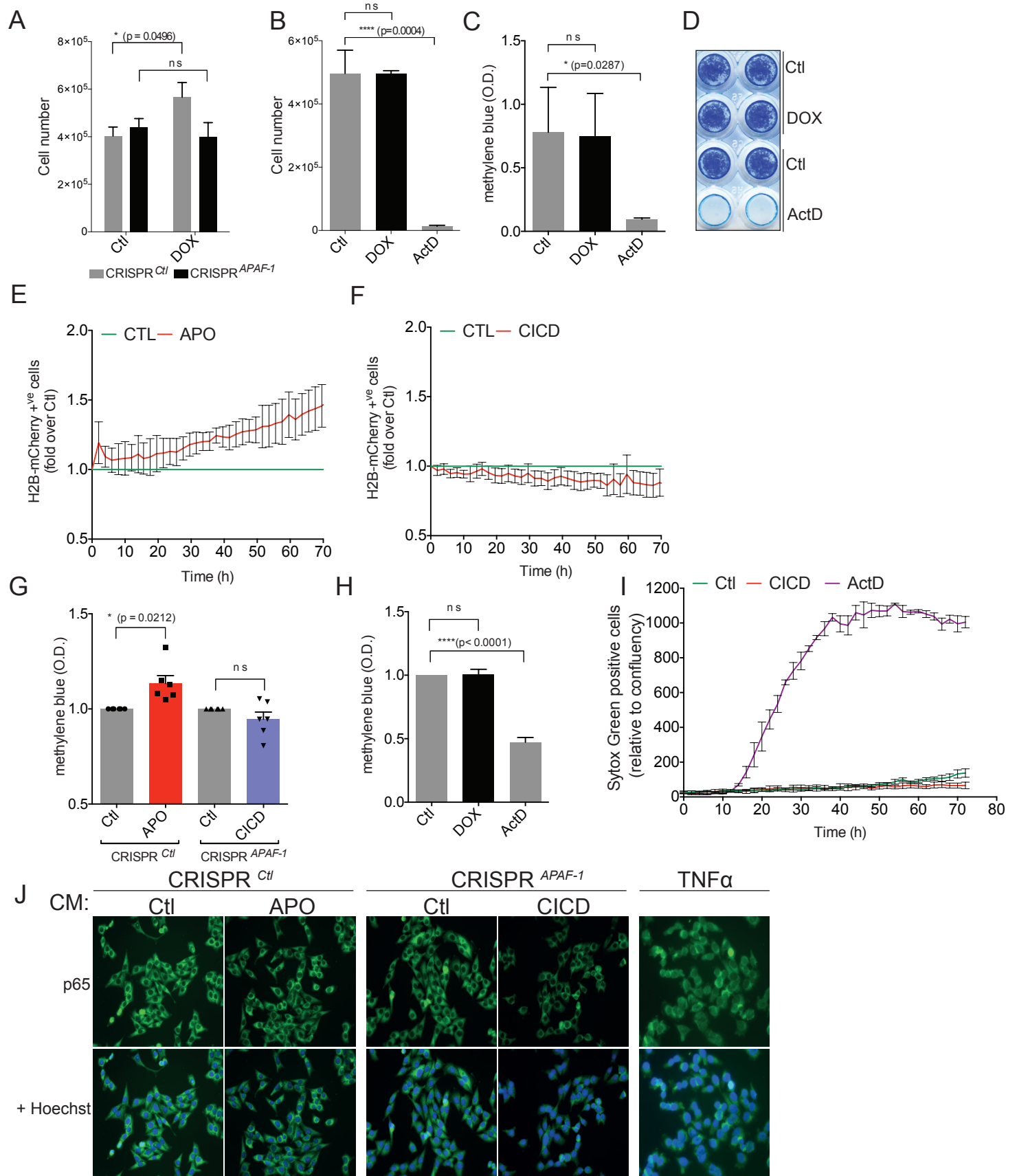

Supplement: Supplementary file 3 — Figure S3. (related to Fig. 2). A. WM115 H2B-mCherry cell counting following 48 h of incubation with either apoptotic or CICD conditioned media. B. Cell counting for assessing the potential effect of doxycycline treatment on WM115 H2B-mCherry cells. C. Cell proliferation of WM115 H2B-mCherry cells in response to doxycycline treatment (1 μg/ml) was assessed by quantifying the optical density of methylene blue staining. D. Same as in C, representative methylene blue-stained cells are shown. E-F. Incucyte analysis for the proliferation of WM239A H2B-mCherry cells grown in the presence of APO (E) or CICD (F) conditioned media. n = 3 independent experiments; mean values +/− s.e.m. G-H. Cell proliferation of WM239A H2B-mCherry cells grown for 48 h in APO or CICD conditioned media was measured by quantifying the optical density (O.D.) of methylene blue staining (G), while in H same approach was done to exclude a potential effect of DOX treatment on cell proliferation. I. Incucyte analysis for the cell death induction (SYTOX Green exclusion) triggered by CICD conditioned media in WM239A H2B-mCherry cells. Actinomycin D treatment (1 μM) is used as positive control for cell death induction. A representative experiment is shown. J Immunofluorescence representative images for the nuclear translocation of p65 in WM115 H2B-mCherry cells grown in presence of apoptotic or CICD conditioned media. Treatment for 3 h with TNFα (20 ng/ml) was used as positive control for p65 nuclear translocation. (PDF 706 kb) [file 12860_2018_164_MOESM3_ESM.pdf]

Figure S4

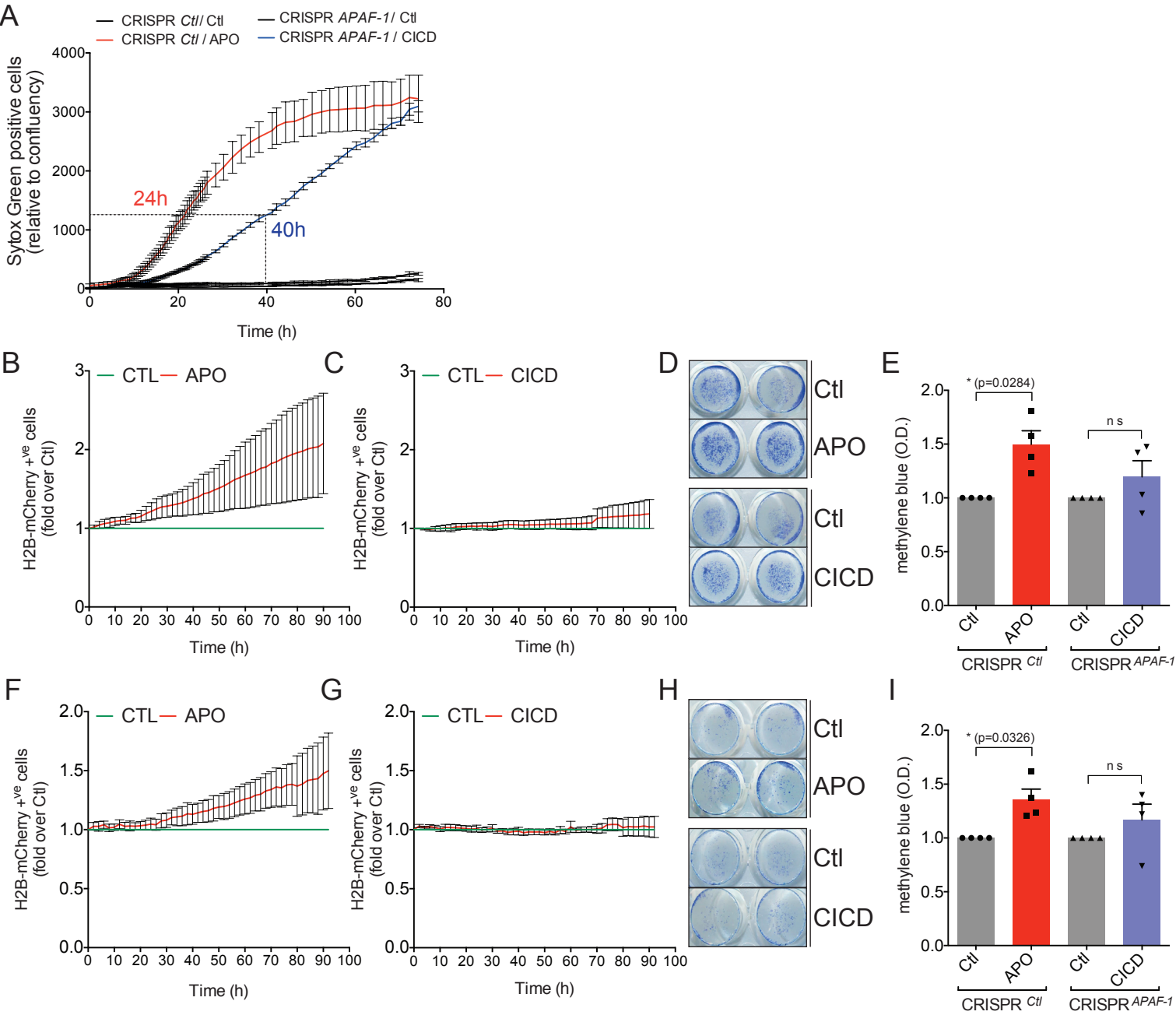

Supplement: Supplementary file 4 — Figure S4. (related to Fig. 2). A. SYTOX Green analysis for the cell death kinetics allowing to identify the 24 and 40 h time frame where apoptosis and CICD, respectively, reach the same extent of execution. A representative experiment is shown. B-C. WM115 H2B-mCherry cells were grown in either apoptotic (obtained at 24 h following doxycycline treatment) (B) or CICD conditioned media (collected 40 h after triggering CICD) (C) and the number of H2B-mCherry positive cells was automatically counted using an Incucyte imager. n = 3 independent experiments; mean values +/− s.e.m. D-E. Representative images of methylene blue staining (D) and the optical density quantification (E) for assessing the proliferation of WM115 H2B-mCherry cells grown as described in B-C. F-I. Same as in B-E, the proliferation of WM239A H2B-mCherry cells is determined by either Incucyte imager or methylene blue staining. For the Incucyte analysis, n = 4–5 independent experiments; mean values +/− s.e.m. (PDF 329 kb) [file 12860_2018_164_MOESM4_ESM.pdf]
